# Surgical Management of Multi-Ligamentous Knee Injuries: Current Concepts and Case Report of a Complex KD-IV Case

**DOI:** 10.3390/reports9020123

**Published:** 2026-04-17

**Authors:** Simone Giusti, Edoardo De Fenu, Simona Cerulli, Ezio Adriani

**Affiliations:** 1 Complex Operational Unit of Sports Medicine and Joint Reconstruction, Fondazione Policlinico Universitario Agostino Gemelli IRCCS, 00168 Rome, Italy; edoardo.defenu@policlinicogemelli.it (E.D.F.); simona.cerulli@policlinicogemelli.it (S.C.); ezioadriani@gmail.com (E.A.); 2 Department of Geriatrics, Orthopaedics, and Rheumatology, Università Cattolica del Sacro Cuore, 20123 Milan, Italy

**Keywords:** knee, multiligament, injury, ACL, PCL, MCL, technique

## Abstract

**Background and Clinical Significance**: Multiligamentous knee injuries (MLKIs) are uncommon but severe injuries associated with instability, neurovascular compromise, and long-term functional impairment. Irreducible knee dislocations are a distinct subgroup requiring urgent intervention because soft-tissue interposition may prevent closed reduction and place the limb at risk of skin necrosis and vascular compromise. This report reviews current concepts in MLKI management and presents a complex KD-IV irreducible knee dislocation treated with a staged surgical strategy. **Case Presentation**: A 56-year-old woman presented 24 h after a skiing injury with a grossly deformed knee, multidirectional instability, and an anteromedial “pucker sign”. Magnetic resonance imaging demonstrated a KD-IV injury with complete rupture of the anterior cruciate ligament, posterior cruciate ligament, and medial collateral ligament, associated with capsular disruption and intra-articular soft-tissue interposition causing irreducibility. Urgent open reduction was performed. The first stage included reduction of the incarcerated capsule, capsular repair, and reconstruction of the posteromedial corner and medial collateral ligament using a semitendinosus autograft. Delayed reassessment at 6 months demonstrated satisfactory stability, minimal residual anterior laxity, and no subjective instability; therefore, anterior cruciate ligament reconstruction was not performed. At final follow-up, the patient had near-full range of motion, no significant valgus instability, and no arthrofibrosis or vascular complications. **Conclusions**: Management of MLKIs should be individualized according to reducibility, soft-tissue condition, neurovascular status, and functional demands. Irreducible KD-IV dislocations with a pucker sign require urgent open reduction. In selected patients, staged reconstruction may reduce postoperative stiffness and allow selective omission of cruciate ligament reconstruction when satisfactory functional stability is achieved.

## 1. Introduction and Clinical Significance

Multiligamentous knee injuries (MLKIs) represent a rare but devastating injury, characterized by disruption of at least two of the major stabilizing structures of the knee, often in association with frank tibiofemoral dislocation, capsular injury, neurovascular compromise, meniscal tears, and chondral damage. These injuries are most commonly associated with high-energy trauma, motor vehicle collisions, and sports injuries, and are frequently accompanied by substantial functional impairment and prolonged recovery periods [1,2]. Despite improvements in diagnostic algorithms, surgical techniques, and rehabilitation protocols, MLKIs continue to pose major challenges because of their heterogeneity, the risk of limb-threatening complications, and the absence of universally accepted treatment algorithms [3,4].

The contemporary management of MLKIs has shifted away from prolonged immobilization and nonoperative care toward early recognition, prompt reduction, careful neurovascular assessment, and individualized surgical reconstruction [1,3,4]. Current consensus supports the use of systematic classification frameworks, including the Schenck classification (Table 1), to characterize injury patterns and guide treatment planning [5,6,7]. However, controversy persists regarding the optimal timing of surgery, the role of staged versus single-stage reconstruction, graft selection, fixation sequence, management of associated posterolateral corner (PLC) injury, and postoperative rehabilitation protocols [2,5,8].

One of the most important early distinctions in MLKI management is whether the dislocation is reducible or irreducible. Irreducible knee dislocations are uncommon but constitute an orthopaedic emergency because interposed soft tissues, including the capsule, medial retinaculum, collateral ligaments, vastus medialis, or meniscus, prevent closed reduction and place the overlying skin at risk of necrosis [9,10,11]. The classic “pucker sign” or “dimple sign” has been described as a pathognomonic finding suggestive of soft-tissue entrapment and irreducibility [9]. Delayed recognition may result in progressive skin compromise, vascular injury, or compartment syndrome, highlighting the importance of urgent open reduction and stabilization in these patients [10,11].

In contrast, the management of reducible MLKIs remains more controversial. Although operative treatment is generally associated with superior functional outcomes compared with conservative management, the ideal timing of reconstruction remains debated [2,3,12]. Historically, acute reconstruction was advocated to restore knee stability and facilitate earlier rehabilitation. However, more recent evidence has suggested that delayed or staged reconstruction may reduce the risk of postoperative stiffness and arthrofibrosis, particularly in complex injury patterns or in the presence of substantial soft-tissue swelling [12,13,14]. In a recent propensity-matched analysis, early reconstruction was associated with increased rates of manipulation under anesthesia and reduced postoperative flexion compared with delayed surgery [12]. Similarly, a systematic review and meta-analysis demonstrated that early surgery and a greater number of injured ligaments were associated with an increased risk of postoperative stiffness [14].

The increasing recognition of arthrofibrosis as a major source of morbidity has significantly influenced contemporary treatment strategies. Arthrofibrosis remains one of the most frequent complications after MLKI reconstruction, yet its definition, diagnosis, and management remain poorly standardized [13]. A recent study identified wide variability in the reported incidence of arthrofibrosis across the literature and the need for standardized postoperative rehabilitation protocols and earlier recognition of stiffness-related complications [13]. Accordingly, modern management increasingly emphasizes balancing restoration of stability with preservation of motion.

The choice between single-stage and staged reconstruction remains another area of ongoing debate. Single-stage reconstruction may provide earlier restoration of knee stability and reduce the number of procedures required, but concerns remain regarding increased operative time, wound complications, and postoperative stiffness [4,8,15]. Conversely, staged reconstruction may allow for recovery of soft tissues, restoration of motion, and more individualized treatment of peripheral and central ligamentous injuries [3,12]. Recent multicenter data from the STaR trial network have shown marked variability in the surgical management of MLKIs across centers, reflecting the absence of a universally accepted treatment algorithm [6].

Graft selection and technical execution are equally important determinants of outcome. Current evidence supports individualized graft choice based on patient age, activity level, tissue quality, injury severity, and surgeon experience [3,8,16]. Autografts may be preferred in younger and more active patients because of their biological incorporation, whereas allografts are commonly used in complex reconstructions involving multiple ligaments, revision procedures, or patients in whom minimizing donor-site morbidity is important [8,16]. In addition, restoration of native anatomy through precise tunnel placement, appropriate graft tensioning, and meticulous fixation remains essential to optimize knee kinematics and long-term function [3,17,18]. The management of associated PLC injury deserves particular attention because unrecognized or inadequately treated PLC deficiency is associated with persistent instability, graft overload, and chronic knee pain [19,20].

Postoperative rehabilitation is increasingly recognized as a cornerstone of successful MLKI management. Contemporary protocols emphasize early protected range of motion, progressive weight-bearing, quadriceps activation, neuromuscular retraining, and criterion-based return to sport [5,21,22]. Early mobilization has been associated with improved motion and a lower risk of arthrofibrosis without compromising graft integrity [16,21,22]. However, return to sport after MLKI remains inconsistent and frequently prolonged. Recent evidence suggests that return to preinjury levels of sport is achieved in only a subset of patients, with many requiring 12 months or longer before return to unrestricted activity [21,23].

Although substantial progress has been made, important gaps remain in the literature regarding the management of irreducible dislocations, the role of staged surgery, the necessity of reconstructing all injured ligaments in every patient, and the long-term consequences of residual laxity. The purpose of this study is therefore to contextualize a complex KD-IV MLKI case within the contemporary literature, with particular emphasis on the management of irreducible dislocation, staged reconstruction strategies, and individualized decision-making regarding cruciate ligament reconstruction.

## 2. Case Presentation

Ethical approval was obtained from our institution, and the study was conducted according to the Helsinki Declaration. Written consent was obtained from the patient to publish the information/image(s) in an online open-access publication. A 56-year-old otherwise healthy female was referred to our center 24 h after a skiing accident. She remembered losing her balance and falling to the ground, with her left knee moving in what she described as an “unnatural” way. She experienced immediate severe pain and, upon trying to bear weight, a sense of “giving way” in her knee. She was attended by the local paramedics, who immediately noted that the knee appeared severely deformed. She was escorted to the local emergency department, where she underwent primary radiographic examinations, which excluded the presence of any fractures around the knee. She was mistakenly diagnosed with a patellar dislocation due to the deformed aspect of her anterior-lateral knee profile and referred to our specialist knee center for further treatment.

**Figure 1:**
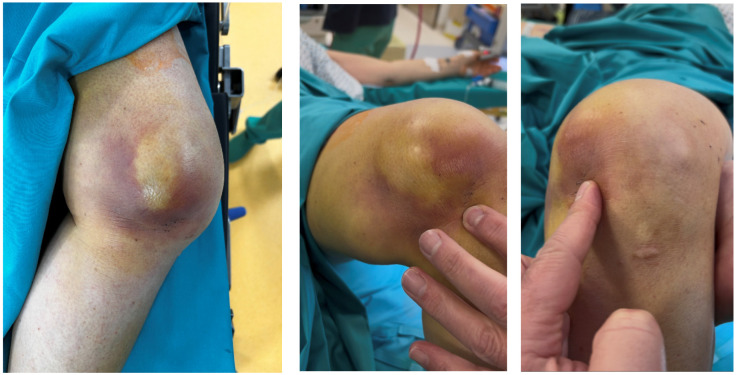
Physical examination revealed the “pucker sign”.

Upon arrival at our institution, her left knee appeared grossly deformed with marked swelling and multi-directional instability. A characteristic “pucker sign” [7,24] (transverse indentation) was noted on the antero-medial aspect of the knee, as shown in Figure 1. Distal pulses were palpable, and no sensory deficits were noted. The popliteal artery was intact on Doppler ultrasound.

**Figure 2:**
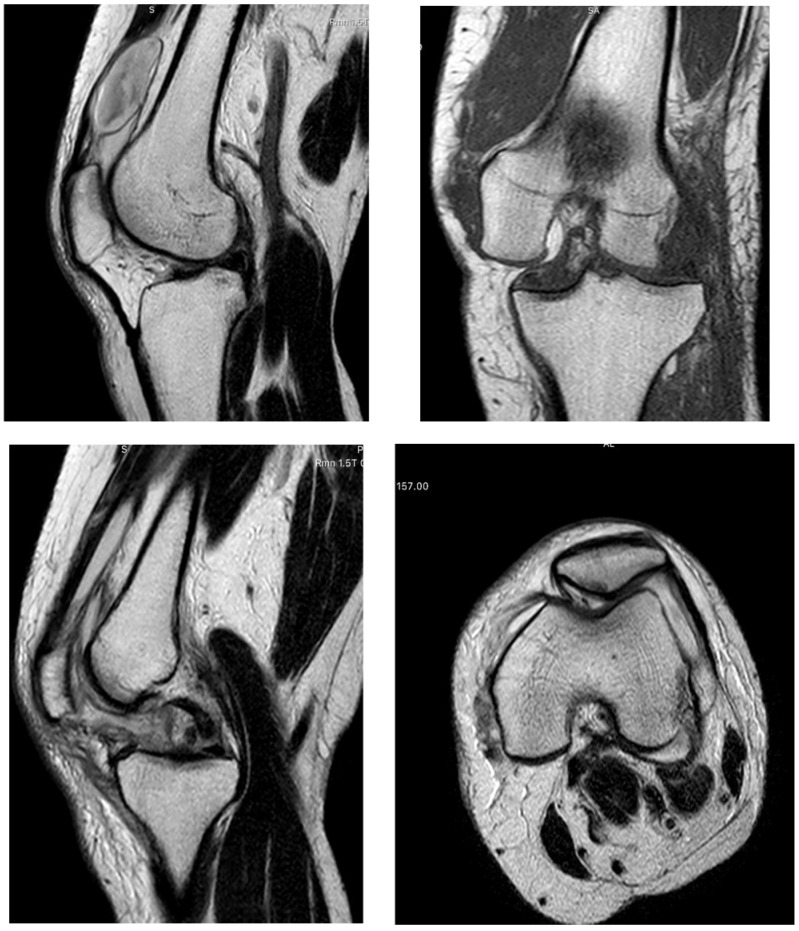
MRI showing extensive soft tissue swelling and effusion, knee dislocation with intra-articular medial capsule subluxation, complete rupture of the ACL, PCL, and MCL, and lateral subluxation of the patella.

She was sent for magnetic resonance imaging (MRI) of her knee (Figure 2), which revealed a dislocated knee joint with complete rupture of the ACL, PCL, and MCL with rupture of the medial articular capsule, which appeared dislocated articularly inside the knee joint, thus making the dislocation irreducible.

A decision to proceed with a two-step surgical reconstruction was made by the team due to the extensive soft-tissue swelling and elevated risk of postoperative arthrofibrosis. The first step of the procedure would consist of the open reduction of the dislocation, followed by surgical management of the “periphery”, including MCL treatment and articular capsule repair.

**Step 1—Periphery**

The patient was placed in the supine position with a tourniquet placed at the root of the left thigh at a pressure of 280 mmHg.

**Figure 3:**
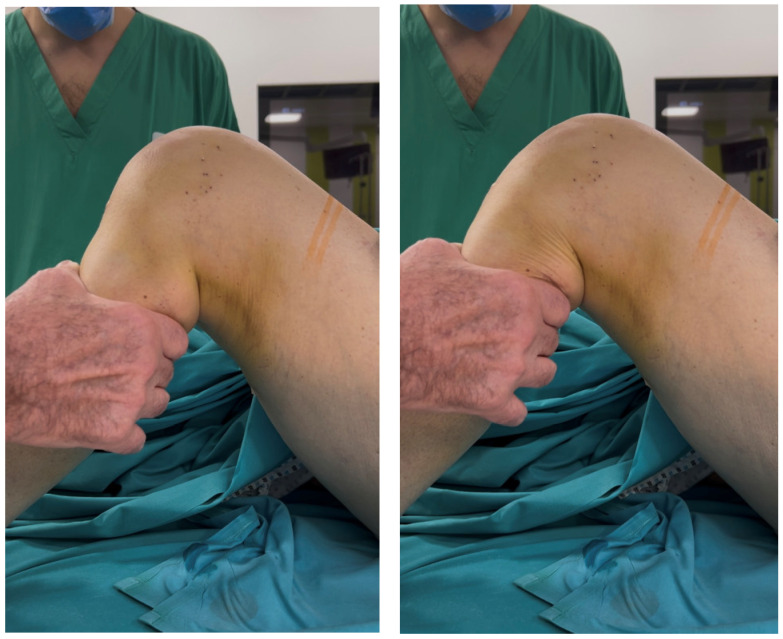
Positive anterior and posterior drawer test.

Examination under anesthesia revealed a + anterior and posterior drawer test and a + valgus thrust test (Figure 3).

**Figure 4:**
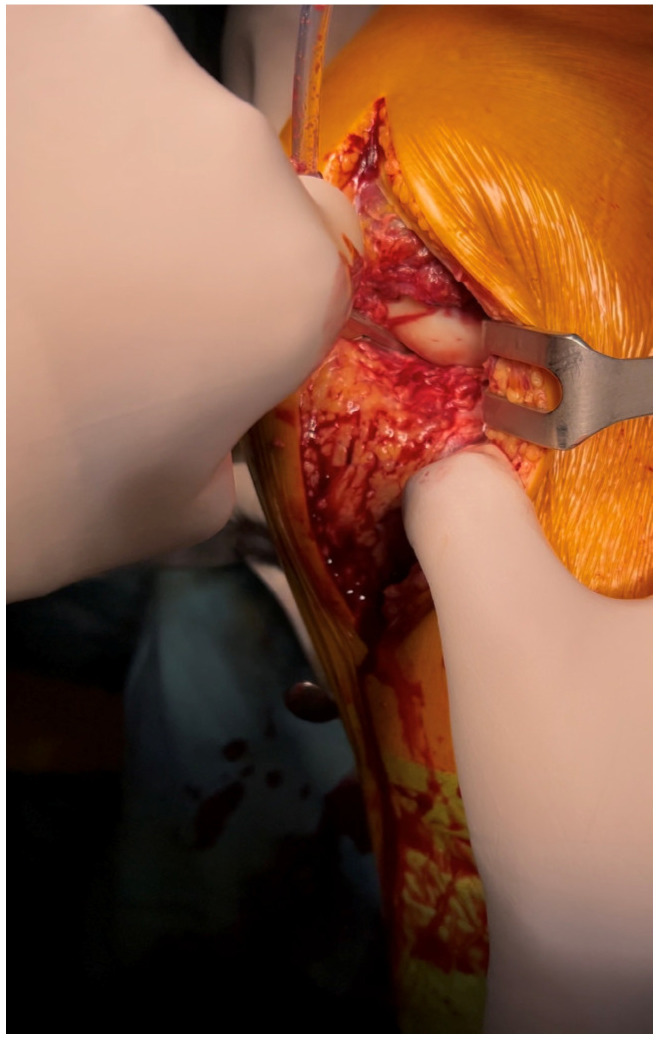
Antero-medial capsular lesion with intra-articular luxation of the capsule.

A longitudinal 15 cm anteroposterior-medial skin incision was performed on the knee. Subcutaneous tissue and fat were carefully incised with accurate coagulation of any small vessels. We identified a complete antero-medial capsular lesion with intra-articular luxation of the capsule (Figure 4).

**Figure 5:**
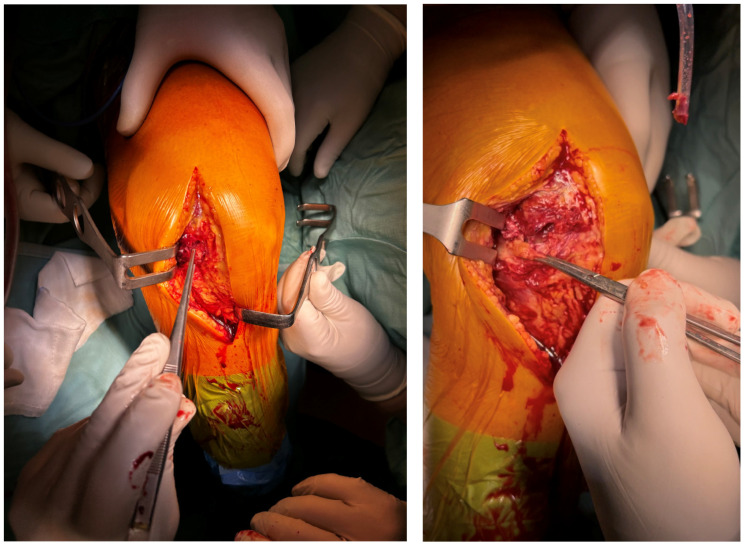
Complete reduction in the antero-medial capsular lesion.

We proceeded to reduce the capsular luxation as shown in Figure 5.

**Figure 6:**
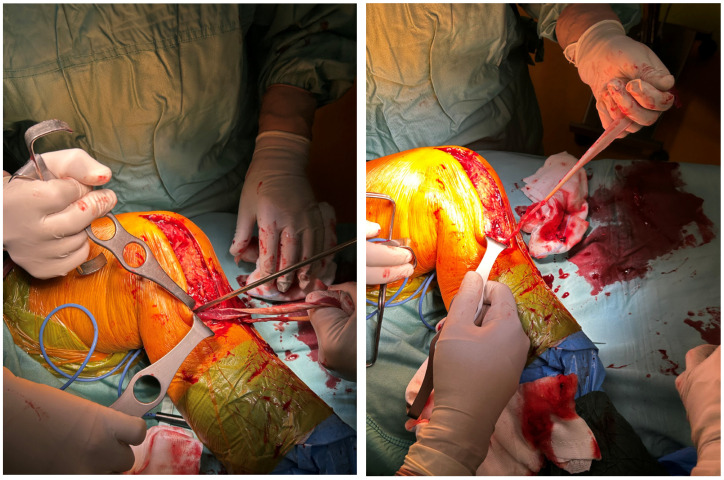
Harvesting of the semitendinosus tendon.

The sartorius fascia was identified and incised, revealing the semitendinosus tendon, which was harvested using a standard technique, whilst retaining its tibial insertion (Figure 6).

**Figure 7:**
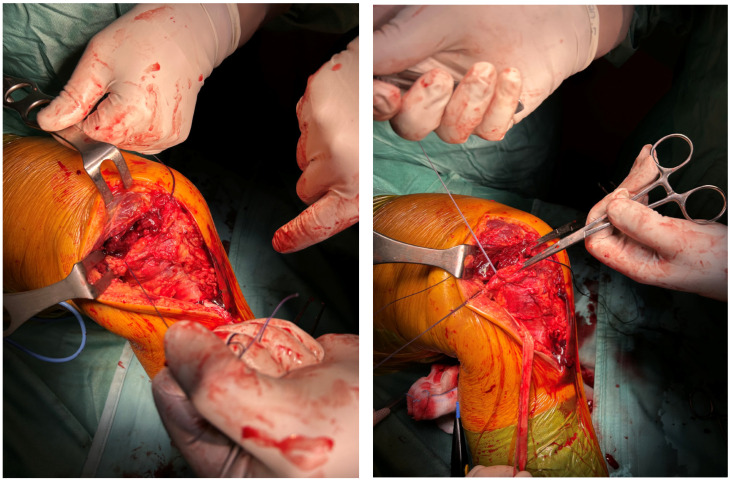
Preparation of the articular capsule with high-resistance sutures.

The articular capsule was subsequently prepared with high-resistance sutures (fiberwire no. 2, Arthrex, Naples, FL, USA), as shown in Figure 7.

**Figure 8:**
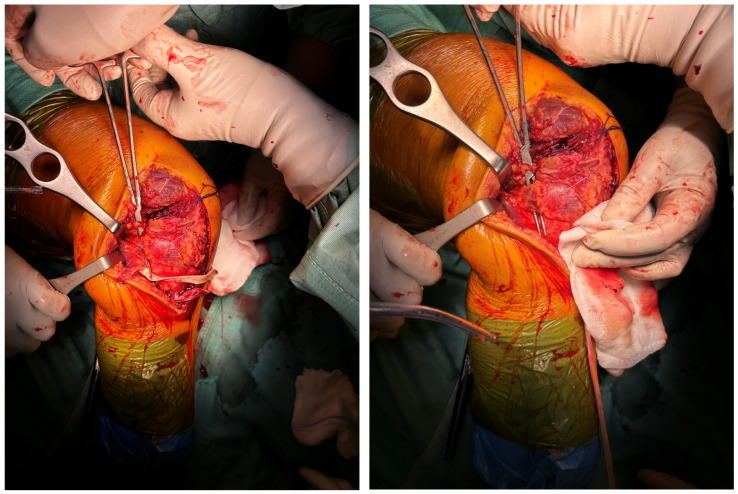
The semitendinosus tendon is threaded underneath the semimembranosus tendon.

The semimembranosus tendon (direct band) was identified through a 2 cm fascial incision, approximately 2 cm distal to the posterior joint line. We then proceeded to thread the free end of the semitendinosus tendon underneath the direct band of the semimembranosus tendon (Figure 8).

**Figure 9:**
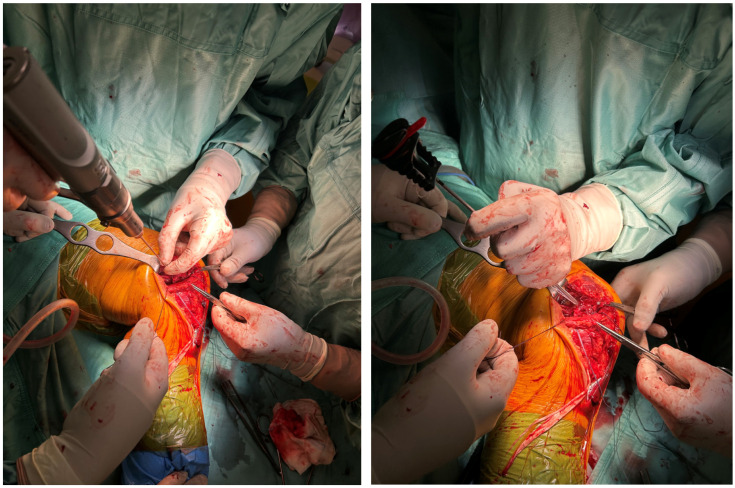
Pre-drilling and insertion of the FiberTak Anchor System.

We proceed to pre-drill the bone immediately behind the medial femoral epicondyle with a 2.4 mm drill. A 2.6 mm FiberTak Knotless Anchor System (Arthrex, Naples, FL, USA) was inserted in the pre-drilled hole (Figure 9).

**Figure 10:**
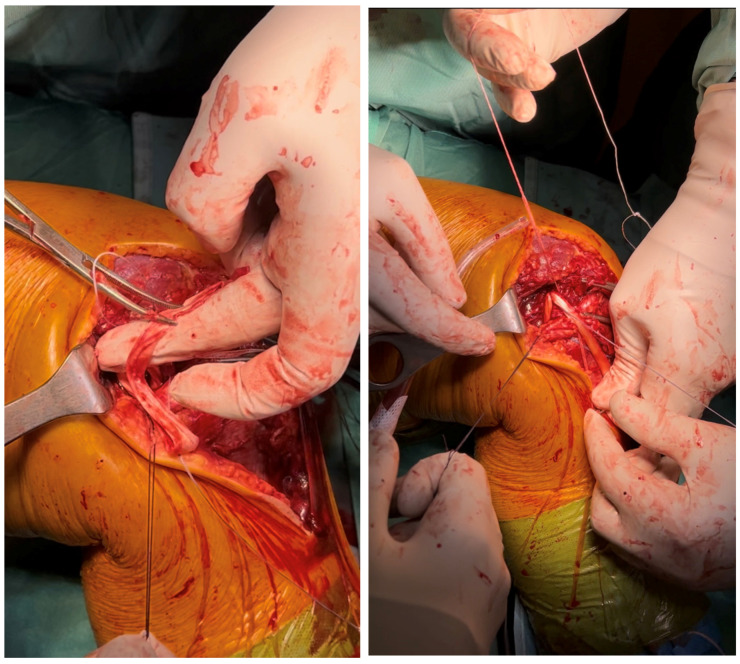
Semitendinosus tendon threading through the buttonhole and fixation to the bone.

The free end of the semitendinosus tendon was then threaded into the buttonhole of the anchor system and fixed to the femoral bone in mid-knee flexion to reconstruct the postero-oblique ligament (POL) (Figure 10).

**Figure 11:**
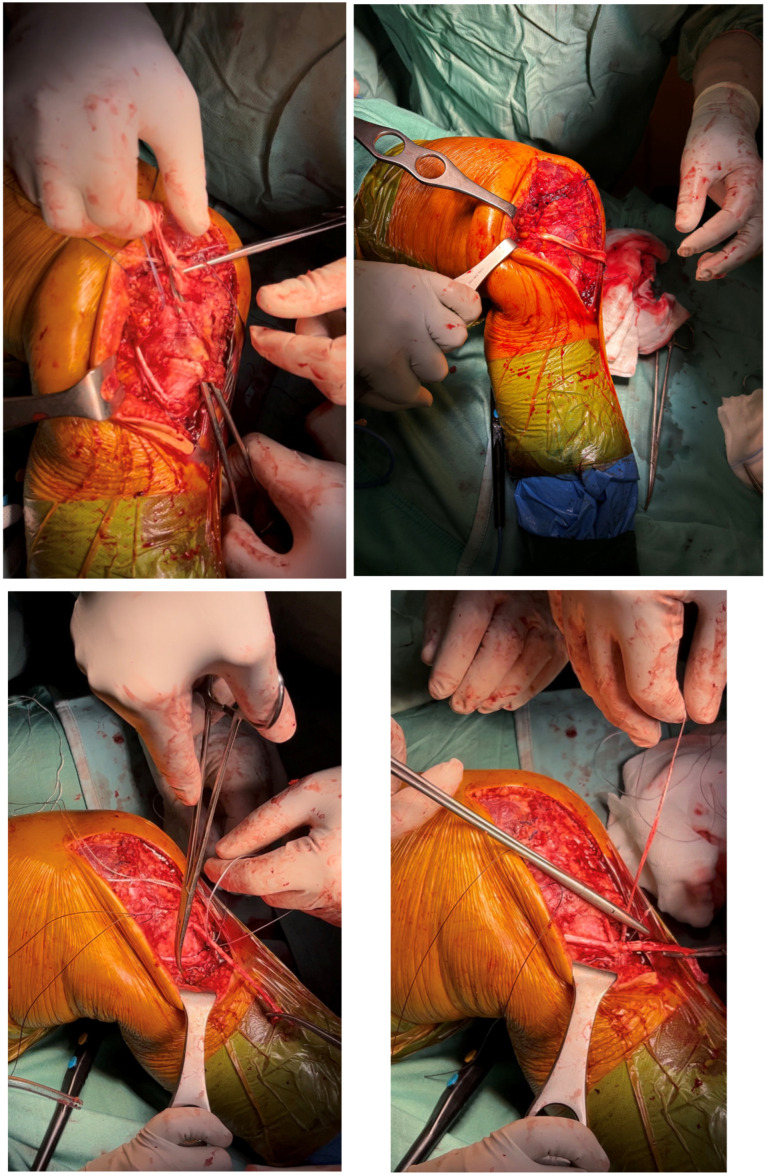
Residual semitendinosus tendon threading underneath the fascia and distal tibial fixation on the MCL footprint with anchor system technique.

The residual free end of the semitendinosus tendon was subsequently threaded back distally underneath the fascial layer and anchored to the tibia on the native MCL insertion at about 45° of knee flexion using the same technique and anchor system used on the femur (Figure 11).

**Figure 12:**
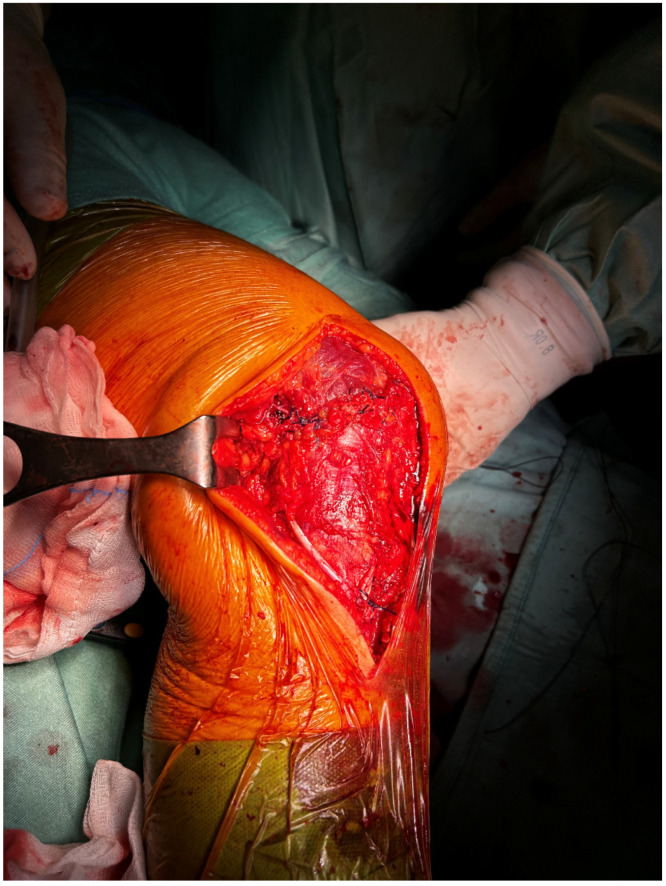
Complete capsular closure with high-resistance sutures.

Finally, a meticulous capsular repair was performed with high-resistance sutures on the previously prepared capsular margins (Figure 12).

**Step 2—Central Pivot Structures (ACL and PCL) assessment**

After 6 months from the peripheral reconstruction, we were prepared to proceed with the reconstruction of the ACL and PCL. However, at this time, we performed an accurate examination that demonstrated optimal healing of the peripheral reconstruction and minimal residual valgus thrust with a—posterior drawer test and a + anterior drawer test, which was, however, well tolerated by the patient. Together with the patient, we decided not to proceed with the reconstruction of the ACL.

At final follow-up, the patient demonstrated satisfactory functional recovery with a stable knee in daily activities. Clinical examination revealed a near-full range of motion (0–110°) with no significant valgus instability and a negative posterior drawer test. The patient reported no subjective instability and returned to normal daily activities without limitations. No complications such as infection, arthrofibrosis, or vascular impairment were observed.

The decision to avoid ACL reconstruction was based on satisfactory clinical stability, absence of functional instability, and patient-specific factors, including age and activity level. Current literature supports selective reconstruction in MLKIs, particularly when acceptable functional outcomes can be achieved without addressing all injured ligaments. Sequence of surgical repair can be found in Table 2.

## 3. Discussion

This case highlights several important principles in the contemporary management of MLKIs. First, the presence of a “pucker sign” strongly suggested an irreducible dislocation caused by soft-tissue interposition and mandated urgent open reduction. This finding is well established in the literature and has consistently been associated with incarceration of medial structures and potential vascular compromise [9,10,11]. Jeevannavar and Shettar first emphasized the clinical significance of the pucker sign as an indicator of irreducibility [9], while more recent studies have further reinforced the need for immediate surgical intervention to prevent skin breakdown and soft-tissue damage [10,11].

In our case, the decision to proceed with staged reconstruction rather than acute comprehensive reconstruction was guided by concerns regarding soft-tissue status, the extent of injury, and the risk of postoperative stiffness. This strategy is supported by a growing body of evidence suggesting that delayed or staged reconstruction may be advantageous in selected patients with severe MLKIs [3,12,14]. A recent paper showed that early reconstruction was associated with significantly higher rates of manipulation under anesthesia and lower postoperative flexion compared with delayed surgery [12]. A similar study found that both early surgery and a greater number of injured ligaments were independently associated with an increased risk of postoperative stiffness [14]. These findings are particularly relevant because arthrofibrosis remains one of the most common and debilitating complications after MLKI reconstruction [13].

The current literature increasingly supports individualized surgical timing rather than a universal early or delayed approach. Patients with vascular injury, open injuries, fractures, irreducible dislocations, or threatened skin require urgent intervention, whereas patients with reducible dislocations and significant swelling may benefit from delayed central ligament reconstruction after restoration of range of motion and soft-tissue recovery [1,4,5,8]. This approach is also consistent with the expert consensus statement, which emphasized the need for patient-specific treatment algorithms based on injury pattern, neurovascular status, and soft-tissue condition [5].

A particularly important feature of the present case was the decision not to reconstruct the ACL after satisfactory restoration of functional stability following peripheral repair and stabilization. This decision reflects an increasing understanding that not all patients will necessitate reconstruction of the ACL. Although restoration of anatomy remains an important goal, treatment should be individualized according to patient age, activity demands, residual instability, and functional outcome [3,8]. In selected lower-demand patients or in those who regain satisfactory stability after treatment of peripheral structures, omission of ACL reconstruction may avoid unnecessary morbidity without compromising functional outcomes.

The importance of peripheral ligament reconstruction, particularly of the PLC, cannot be overstated. PLC deficiency is a well-recognized cause of persistent instability and failure of cruciate ligament reconstruction [19,20]. Several studies have emphasized that accurate recognition and anatomic reconstruction of the PLC are essential to restore rotational stability and prevent overload of reconstructed cruciate grafts [19]. Similarly, systematic review data have demonstrated that combined cruciate and PLC injuries require careful surgical planning and comprehensive treatment of all deficient structures to optimize outcomes [20].

Technical considerations also remain critical in MLKI reconstruction. Late studies have highlighted substantial variability in graft selection, tunnel placement, fixation sequence, and graft tensioning protocols [16,17,18]. While biomechanical data suggest that there may be no major difference between ACL-first and PCL-first fixation in single-stage reconstruction [18], heterogeneity persists in clinical practice [17]. In the absence of definitive comparative evidence, surgical strategy should be individualized and guided by the dominant instability pattern, tissue quality, and surgeon experience.

Functional outcomes after MLKI reconstruction are generally acceptable but remain inferior to those reported after isolated ligament injury. Prospective data have shown meaningful improvements in patient-reported outcome measures after multiligament reconstruction, although persistent pain, stiffness, residual laxity, and radiographic osteoarthritis remain common [2,24,25]. Similarly, long-term follow-up studies have demonstrated that although many patients return to work and recreational activity, return to high-level sport is less predictable, with many patients unable to achieve their preinjury level of competition [2,21,23]. Pre-operative counselling for all patients is mandatory to generate realistic expectations for recovery.

Complication rates after MLKI surgery remain substantial; smoking, planned staged procedures, and greater injury severity are common risk factors for complications following surgical management [26]. A recent paper reported high rates of early postoperative complications, with wound problems, infection, arthrofibrosis and venous thromboembolism as the most common [27]. These findings reinforce the need for close postoperative monitoring.

Our study has several limitations. It reflects the experience of a single case and therefore cannot establish causality or define an optimal treatment algorithm. In addition, the absence of long-term follow-up limits assessment of residual instability, osteoarthritis progression, and delayed functional decline. Nevertheless, the case illustrates several key principles relevant to the management of complex MLKIs and supports an individualized, staged approach in selected patients with irreducible dislocation and high risk of postoperative stiffness.

## 4. Conclusions

MLKIs remain among the most challenging injuries in orthopaedics because of their complexity, variability, and potential for severe functional impairment. Prompt recognition, early vascular assessment, and differentiation between reducible and irreducible dislocations are essential to optimize outcomes.

Irreducible dislocations, particularly those associated with a pucker sign or threatened skin, should be considered surgical emergencies requiring urgent open reduction because delayed management may result in skin necrosis, vascular compromise, and progressive soft-tissue injury [9,10,11].

For reducible injuries, management should be individualized according to injury pattern, soft-tissue condition, neurovascular status, patient expectations, and risk of postoperative stiffness. In complex KD injuries, staged reconstruction may offer important advantages by allowing recovery of motion and soft tissues before cruciate reconstruction [12,13,14]. Furthermore, reconstruction of every structure may not be necessary in all patients if satisfactory functional stability can be achieved through selective treatment of the dominant instability pattern.

Successful MLKI management depends on meticulous surgical technique, accurate treatment of peripheral structures such as the PLC, and structured postoperative rehabilitation emphasizing early motion and criterion-based progression [5,20,22,23]. Despite improvements in treatment, complication rates remain high and long-term outcomes remain variable.

Future research should focus on prospective comparative studies evaluating surgical timing, graft selection, rehabilitation strategies, and the role of selective versus comprehensive ligament reconstruction. Greater standardization of definitions, outcome measures, and rehabilitation protocols will be essential to improve evidence quality and guide management in this challenging patient population.

## Figures and Tables

**Table 1:** Schenck’s knee dislocation (KD) classification.

Schenck Knee Dislocation (KD) Classification	Description
**KD-I**	Injury to a single ligament (ACL or PCL)
**KD-II**	Injury to both ACL and PCL
**KD-III**	Injury to ACL and PCL with additional medial (M) or lateral (L) injury: MCL (KD-III-M) or FCL (KD-III-L)
**KD-IV**	Injury to both cruciate and collateral ligaments (ACL, PCL, MCL, FCL)
**KD-V**	Knee fracture-dislocation

ACL, anterior cruciate ligament; FCL, fibular collateral ligament; KD, knee dislocation; MCL, medial collateral ligament; PCL, posterior cruciate ligament.

**Table 2:** Sequence of repair and reconstruction in the presented case.

Step	Structure Addressed	Procedure Performed	Rationale
1	Medial capsule	Open reduction and capsular repair	Removal of intra-articular interposition causing irreducibility
2	Medial structures (POL/MCL)	Semitendinosus autograft reconstruction with anchor fixation	Restoration of medial stability and protection of the central pivot
3	Peripheral stabilization	Soft tissue balancing and layered closure	Reduction in residual instability and protection of repair
4	Central pivot (ACL/PCL)	Delayed reassessment (no ACL reconstruction performed acutely)	Avoid overtreatment to avoid the risk of arthrofibrosis

## Data Availability

The original contributions presented in the study are included in the article, further inquiries can be directed to the corresponding author.
